# Supplementary material for: Effects of disputes and easement violations on the cost-effectiveness of land conservation
Source: PeerJ. 2015 Aug 13;3:e1185. doi: 10.7717/peerj.1185 (PMC4581774; doi:10.7717/peerj.1185)
Supplement: Appendix S1 — This supplementary material includes two Appendices. In Appendix S1 we include a figure of the Georgia Basin of British Columbia, Canada, highlighting the study region. In Appendix S2 we provide the R script that we used for our analysis and simulations. [file peerj-03-1185-s001.docx]

PeerJ

Schuster, R. and Arcese, P. Efficient routes to land conservation given risk of covenant failure.

**Supplementary Information**

This supplementary material includes two Appendices. In Appendix S1 we include a figure of the Georgia Basin of British Columbia, Canada, highlighting the study region. In Appendix S2 we provide the R script that we used for our analysis and simulations.

**Appendix S1:** Coastal Douglas Fir (CDF) ecological zone showing the study area in red.

**
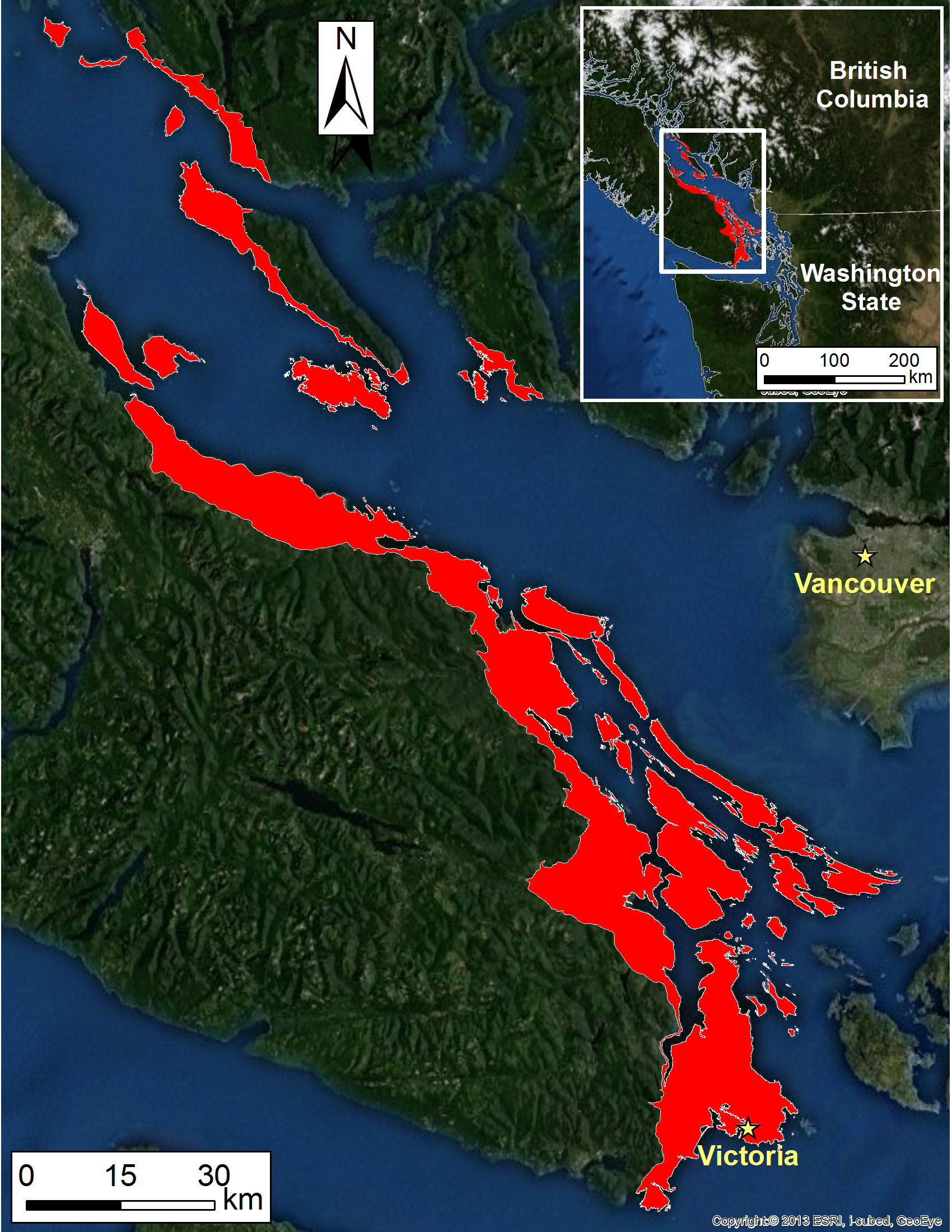
**

**Appendix S2** Simulation and Analysis R code.

In this appendix, we provide the R code for conducting our simulations and analysis used in the paper. R v.3.0.2 (R Core Team 2013) was used and custom distributions were parameterized using package distr v.2.5.2 (Ruckdeschel et al. 2006).

R Core Team. 2013. R: A Language and Environment for Statistical Computing.

Ruckdeschel, P., M. Kohl, T. Stabla, and F. Camphausen. 2006. S4 Classes for Distributions. R News **6**:2–6.

##############################################################################

### Setup Covenant analysis (run the Marxan scenario)

### Richard Schuster (mail@richard-schuster.com

### 2013, Sept 25

##############################################################################

library(Rmarxan2)

setwd("D:\\R_files\\13_09_25_Ch3_analytical_framework_setup")

cad <- read.csv("Single_Poly_post_cov_top_Land_value_IDW_10_groups_R.csv")

# area adjustment to have it in acres

cov.cst <- data.frame(ID=cad$ID, AREA=cad$AREA, ACR=cad$AREA * 0.000247105, COST=cad$CALC_TOTAL)

# fixed costs

#Land owner

LO.legal <- 300

LO.finadv <- 300

LO.registr <- 200

LO.endow <- 10000

#Covenant Holder

CH.legal <- 4000

fixed.all <- LO.legal + LO.finadv + LO.registr + LO.endow + CH.legal

# cost vary with property area

# area unit used is acre

LO.bas.b0 <- -2185.30978

LO.bas.b1 <- 1957.45823

LO.bas.min <- 1000

LO.app.b0 <- 0

LO.app.b1 <- 1957.45823

LO.app.min <- 1500

LO.surv.b0 <- 300

LO.surv.b1 <- 1957.45823

LO.surv.min <- 1000

cov.cst$COV.fix <- fixed.all

cov.cst$COV.bas <- ifelse((LO.bas.b0 + LO.bas.b1 * log(cov.cst$ACR)) > LO.bas.min,

(LO.bas.b0 + LO.bas.b1 * log(cov.cst$ACR)),LO.bas.min)

cov.cst$COV.app <- ifelse((LO.app.b0 + LO.app.b1 * log(cov.cst$ACR)) > LO.app.min,

(LO.app.b0 + LO.app.b1 * log(cov.cst$ACR)),LO.app.min)

cov.cst$COV.surv <- ifelse((LO.surv.b0 + LO.surv.b1 * log(cov.cst$ACR)) > LO.surv.min,

(LO.surv.b0 + LO.surv.b1 * log(cov.cst$ACR)),LO.surv.min)

cov.cst$COV.comb <- rowSums(cov.cst[,4:7], na.rm=T)

##############################################################################

# standard repeat costs

# Monitoring (to see if covenant is intact)

# repeat rate: 1/1 year

# rate used: NCC charge (from Management cost workshop)

mon <- 758

# Staff cost (work done to reply to Land owner request)

# repeat rate: 1/5 years

# rate used: NCC charge (from Management cost workshop)

# presented in yearly portion:

staff <- mon/5

tt <- cov.cst$COV.comb + 50*(mon+staff)

marxan.covenant.pu <- data.frame(id=cov.cst$ID, cost=cov.cst$COV.comb, status=0)

write.csv(marxan.covenant.pu, "input/Cadaster_pu_cost_Covenant.csv", row.names = FALSE)

indir=getwd()

spf <- 3

nitns <- 10000000

# Covenant Marxan costs

puC="Cadaster_pu_cost_Covenant.csv"

# Acquisition Marxan costs

puA="Cadaster_pu_cost_IDW_new.csv"

puvsp="Cadaster_puvsp_no_C_IDW_new_beta_score.csv"

spec="Cadaster_spec_BETA.csv"

#bound="Cadaster_bound_100m_buff.csv"

spffr <- read.csv((file=sprintf("input/%s",spec)))

spffr[spffr$name=="BETA",]$prop <- 0.2 #Set target

write.csv(spffr, sprintf("input/%s",spec), row.names = FALSE)

################################################################################

# Acquisition Marxan run

setwd("./Marxan_Acquisition")

outdir=getwd()

marx.acqu <- marxan(pu=puA,

puvsp=puvsp,

spec=spec,

# bound=bound,

spf=spf, nreps=1000, nitns=nitns, scenname="Acqu.T0.2",

indir=indir,outdir=outdir)

setwd("..")

################################################################################

save.image("Covenants_Acqu_1000_runs.RData")

#pre-emptive enforcement, and damage enforcement

spd <- function (years, cov.frame, dispute, rD1, pu, mon, staff,PE=T,DE=T, rand=F){

for (ii in 2:years){

for (pu in 1:length(cov.frame[,1])){

if (runif(1) > dispute) {

#cost of dispute

dis.cst <- rD1(1) #disp.cost$cost[which.min(disp.cost$prb < runif(1))]

#dis.cst <- 100000*rexp(1,rate=0.5)

if (rand == TRUE) {

alter <- runif(1,-1,1)

} else {

alter <- 0

}

if (DE == TRUE && alter <= 0){

#metric where bd.loss is losely dependent on dis.cst

init.bd.loss2 <- dis.cst / 400000

bd.loss2 <- init.bd.loss2 + rnorm(1,0,0.05)

bd.loss2 <- ifelse(bd.loss2 < 0, 0, ifelse(bd.loss2 > 1, 1, bd.loss2))

cov.frame[pu,4+years+ii] <- cov.frame[pu,4+years+ii-1] * (1 - bd.loss2)

}

else {

cov.frame[pu,4+years+ii] <- cov.frame[pu,4+years+ii-1]

}

if (PE == TRUE && alter >= 0){

# add dispute cost to covenant cost + set minimum cost to $1000

min.cst <- 1000

dis.cst <- ifelse(dis.cst<min.cst,min.cst,dis.cst)

cov.frame[pu,4+ii] <- cov.frame[pu,4+ii-1] + dis.cst

}

else {

cov.frame[pu,4+ii] <- cov.frame[pu,4+ii-1] + (mon + staff)

}

}

else {

# if there is no dispute carry costs forward but add yearly costs

cov.frame[pu,4+ii] <- cov.frame[pu,4+ii-1] + (mon + staff)

# if there is no dispute carry biodiversity values forward

cov.frame[pu,4+years+ii] <- cov.frame[pu,4+years+ii-1]

}

}

}

return(as.data.frame(cov.frame))

}

# setup and Marxan runs in folder:

# 13_09_25_Ch3_analytical_framework_setup

library(distr)

setwd("D:\\R_files\\15_05_08_Ch3_PeerJ")

load("Covenants_Acqu_1000_runs_red.RData")

rm(list=setdiff(ls(), c("marx.acqu","spd")))

#marx.acqu slots

# ssoln

# best

# run

# sums

# mv

# data frame including

# ID

# AREA

# CALC_TOTAL

# Carbon metrics (StC_AWS, SeqC_AWS)

# Biodiv metrics (both AWM and AWS for OF, SAV, BETA)

cad <- read.csv("Polygon_level_Area_Carbon_Biodiv_values.csv")

# area adjustment to have it in acres

cov.cst <- data.frame(ID=cad$ID, AREA=cad$AREA, ACR=cad$AREA * 0.000247105, COST=cad$CALC_TOTAL)

# fixed costs

#Land owner

LO.legal <- 300

LO.finadv <- 300

LO.registr <- 200

LO.endow <- 10000

#Covenant Holder

CH.legal <- 4000

fixed.all <- LO.legal + LO.finadv + LO.registr + LO.endow + CH.legal

# cost vary with property area

# area unit used is acre

LO.bas.b0 <- -2185.30978

LO.bas.b1 <- 1957.45823

LO.bas.min <- 1000

LO.app.b0 <- 0

LO.app.b1 <- 1957.45823

LO.app.min <- 1500

LO.surv.b0 <- 300

LO.surv.b1 <- 1957.45823

LO.surv.min <- 1000

cov.cst$COV.fix <- fixed.all

cov.cst$COV.bas <- ifelse((LO.bas.b0 + LO.bas.b1 * log(cov.cst$ACR)) > LO.bas.min,

(LO.bas.b0 + LO.bas.b1 * log(cov.cst$ACR)),LO.bas.min)

cov.cst$COV.app <- ifelse((LO.app.b0 + LO.app.b1 * log(cov.cst$ACR)) > LO.app.min,

(LO.app.b0 + LO.app.b1 * log(cov.cst$ACR)),LO.app.min)

cov.cst$COV.surv <- ifelse((LO.surv.b0 + LO.surv.b1 * log(cov.cst$ACR)) > LO.surv.min,

(LO.surv.b0 + LO.surv.b1 * log(cov.cst$ACR)),LO.surv.min)

# combined initial covenant cost of each parcel in the CDF

# does not include any reoccurring costs (they are calculated below

cov.cst$COV.comb <- rowSums(cov.cst[,5:8], na.rm=T)

##############################################################################

# standard repeat costs

# Monitoring (to see if covenant is intact)

# repeat rate: 1/1 year

# rate used: NCC charge (from Management cost workshop)

mon <- 758

# Staff cost (work done to reply to Land owner request)

# repeat rate: 1/5 years

# rate used: NCC charge (from Management cost workshop)

# presented in yearly portion:

staff <- mon/5

################################################################################

################################################################################

## MARXAN RUNS COMPLETE

## START COVENANT UNCERTAINTY ANALYSIS

################################################################################

################################################################################

################################################################################

#Setup from Rissman

#Data from Rissman 2010 Fig. 1

inc.rate <- data.frame(year=c(seq(1989,2007,1)),

issues=c(1,1,NA,1,NA,NA,2,1,NA,3,2,2,5,1,3,3,4,8,5))

fm2 <- glm(issues~year, data=inc.rate, family=quasi(link="log",variance="constant"))

fm2.lin <- lm(issues~year, data=inc.rate)

# for logistic growth

# Formula: N(t) = CC * N0 * exp(rr*t) / (CC + N0 * (exp(rr*t) - 1))

# growth rate rr

rr <- coef(fm2.lin)[[2]]

# Carrying Capacity CC

CC <- 50

# "Population" at year 2013

N0 <- predict(fm2.lin,newdata=data.frame(year=c(2013)))

#Data from Rissman 2010 Fig. 2

costs <- data.frame(value=c(5000,seq(10000,100000,10000),300000,400000),

incidents=c(12,7,9,4,1,1,2,2,1,1,1,1,1))

fm1 <- nls(incidents ~ a*value^b, data=costs,start = list(a = 2555, b = -0.655))

sc <- coef(fm1)[[1]]

pw <- coef(fm1)[[2]]

f <- function(x) {

return(sc*x^pw)

}

#dispute cost range

bins <- seq(1000,400000,500)

pred <- f(bins)

# length of segments

pred.1 <- pred/sum(pred)

#put pred.1 lengths on a vector between 0 and 1

pred.2 <- vector()

pred.2[1]<- pred.1[1]

for (ii in 2:length(pred.1)){

pred.2[ii] <- pred.2[ii-1] + pred.1[ii]

}

#create lookup data.frame for pred.2 vector values that correspond to bins

disp.cost <- data.frame(prb=pred.2,cost=bins)

D1 <- DiscreteDistribution (supp = bins , prob = pred.1)

dD1 <- d(D1) ## D1ensity function

pD1 <- p(D1) ## D1istribution function

qD1 <- q(D1) ## Quantile function

rD1 <- r(D1) ## Random number generation

### End setup from Rissman 2010

################################################################################

# check if all nreps runs met their target

summary(marx.acqu$sums[[2]]$MPM)

summary(marx.acqu$sums[[2]]$Shortfall)

# check acquisition reserve system cost

hist(marx.acqu$sums[[2]]$Cost)

summary(marx.acqu$sums[[2]]$Cost)

mean(marx.acqu$sums[[2]]$Cost)

sd(marx.acqu$sums[[2]]$Cost)

# assume all purchases are cost + 15%, and that all the

# proceeds of the 15% put into trust are spent in land management to

# maintain the value of the property

marx.acqu$sums[[2]]$Cost <- marx.acqu$sums[[2]]$Cost * 1.15

total.beta <- sum(cad$BETA_AWS)

runs <- marx.acqu$run

mv <- marx.acqu$mv

sums <- marx.acqu$sums

#!!!!!!!!!!!!!!!!!!!!!!!!!!!!

# NEEDS CHANGE FOR FINAL RUNS

years <- 100

nruns <- 100

#dispute rate: 2.8/1000 per year

# 2.8 is the average dispute rate from Rissman 2010

dispute <- 1 - (0.28/1000)

run.sum <- data.frame(ID=0,Init.cost=0,Cost.no.disp=0,Init.Beta=0)

run.sum[sprintf("cost.y%02d",seq(1,years,1))] <- 0

run.sum[sprintf("Beta.y%02d",seq(1,years,1))] <- 0

run.sum.PE <- run.sum.DE <- run.sum.PE.DE.alter <- run.sum

for (rn in 2: (nruns + 1)){

#extract polygon ID's that were selected by Marxan run

#cad$ID[ex==1]

#extract covenant costs that were selected by Marxan run

#cov.cst$COV.comb[ex==1]

#extract BETA values that were selected by Marxan run

#cad$BETA_AWS[ex==1]

#extract run to work with

ex <- runs[rn]

# cost framework for the covenants per run

cov.frame <- data.frame(ID=cad$ID[ex==1], Init.cost=cov.cst$COV.comb[ex==1],

Cost.no.disp=cov.cst$COV.comb[ex==1] + years * (mon + staff), Init.Beta=cad$BETA_AWS[ex==1])

cov.frame[sprintf("cost.y%02d",seq(1,years,1))] <- NA

cov.frame[sprintf("Beta.y%02d",seq(1,years,1))] <- NA

#setup cost and beta for year 1

cov.frame[,5] <- cov.frame[,2]

cov.frame[,5+years] <- cov.frame[,4]

dum <- as.matrix(cov.frame)

cov.frame <- spd (years, dum, dispute, rD1, pu, mon, staff,PE=T,DE=T)

cov.frame.PE <- spd (years, dum, dispute, rD1, pu, mon, staff,PE=T,DE=F)

cov.frame.DE <- spd (years, dum, dispute, rD1, pu, mon, staff,PE=F,DE=T)

cov.frame.PE.DE.alter <- spd (years, dum, dispute, rD1, pu, mon, staff,PE=T,DE=T, rand=T)

#summarize cov.frame rows

#tempR <- sapply(cov.frame,sum)

run.sum[rn-1,] <- sapply(cov.frame,sum)

run.sum.PE[rn-1,] <- sapply(cov.frame.PE,sum)

run.sum.DE[rn-1,] <- sapply(cov.frame.DE,sum)

run.sum.PE.DE.alter[rn-1,] <- sapply(cov.frame.PE.DE.alter,sum)

#run ID into run.sum data frame

run.sum[rn-1,1] <-run.sum.PE[rn-1,1] <-run.sum.DE[rn-1,1] <- run.sum.PE.DE.alter[rn-1,1] <- sprintf("run_%04d",rn-1)

#tempR[1] <- sprintf("run_%04d",rn-1)

#run.sum <- rbind(run.sum, tempR)

}
